# Preparation of the intact rodent organ of Corti for RNAscope and immunolabeling, confocal microscopy, and quantitative analysis

**DOI:** 10.1016/j.xpro.2021.100544

**Published:** 2021-05-24

**Authors:** Daniel O.J. Reijntjes, J. Lukas Breitzler, Dora Persic, Sonja J. Pyott

**Affiliations:** 1 The Center for Hearing and Balance, Department of Otolaryngology-Head and Neck Surgery, The Johns Hopkins University School of Medicine, Baltimore, MA 21201, USA; 2 Department of Otorhinolaryngology-Head and Neck Surgery, University Medical Center Groningen, University of Groningen, 9713GZ Groningen, the Netherlands

**Keywords:** Microscopy, Antibody, In Situ Hybridization, Neuroscience, Molecular/Chemical Probes

## Abstract

This protocol describes the preparation of the mouse organ of Corti for RNAscope, immunolabeling, confocal microscopy, and quantitative image analysis to examine transcript and protein localization, sensory hair cells, and synapses. This protocol can be applied to mice and other rodents (juvenile and adult) and can be adapted for other techniques, including electrophysiology and RNA sequencing. This protocol features minimal tissue processing to preserve viability for downstream assays, while isolating the organ of Corti is the most challenging step.

For additional details on the use and execution of this protocol, please refer to McLean et al. (2009); Schuth et al. (2014); Lingle et al. (2019); Pyott et al. (2020).

## Highlights

• Instructions to prepare the mouse organ of Corti for RNAscope and immunolabeling
• Guidelines for optimal imaging using epifluorescent and confocal microscopy
• Workflow and custom code to quantify hair cell and synapse number and architecture
• Applications/modifications for other animal models and additional techniques

## Graphical abstract

**Figure:**
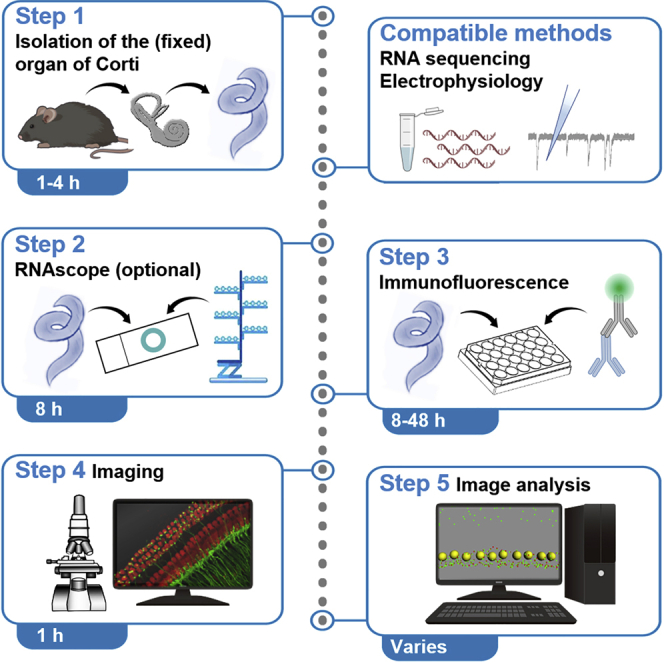

## Before you begin

**Timing: 2 h**

Very little preparation is necessary. Necessary reagents should be made in advance. Dissecting equipment should be assembled. Access to necessary microscopes and computer workplaces should be secured. (See details provided in the materials and equipment section below).

***Note:*** This protocol has been used to isolate the sensory epithelium of various rodents (e.g., Barone et al. 2019), including aged rodents. The protocol has been adapted for use on the isolated vestibular sensory epithelia (e.g., Sadeghi et al. 2014, Schuth et al. 2014, Greguske et al. 2021).

## Key resources table

**Table:** 

REAGENT or RESOURCE	SOURCE	IDENTIFIER
**Antibodies**^a^		

Mouse monocolonal (IgG1) anti-CTBP2 (1:400)	BD Biosciences	612044
Mouse monocolonal (IgG2A) anti-GluR2 (1:300)	Millipore	MAB397
Rabbit polyclonal anti-GluR2/3 (1:300)	Millipore	AB1506
Rabbit polyclonal anti-Calretinin (1:500)	Millipore	AB5054
Rabbit polyclonal anti-Prestin (1:2000)	Dr. Mary Ann Cheatham (Northwestern University, IL, USA) (Zheng et al. 2006)	N/A
Rabbit polyclonal anti-MYO6 (Myosin VI, KA-15) (1:500)	Sigma-Aldrich	M5187
Goat anti-Mouse IgG1 Cross-Adsorbed Secondary Antibody, Alexa Fluor 488 (1:500)	Thermo Fisher Scientific	A-21121
Goat anti-Rabbit(H+L) Cross-Adsorbed Secondary Antibody, Alexa Fluor 488 (1:500)	Thermo Fisher Scientific	A-11008
Goat anti-Rabbit IgG (H+L) Cross-Adsorbed Secondary Antibody, Alexa Fluor 568 (1:500)	Thermo Fisher Scientific	A-11011
Goat anti-Mouse IgG2a Cross-Adsorbed Secondary Antibody, Alexa Fluor 647 (1:500)	Thermo Fisher Scientific	A-21241

**Chemicals, peptides, and recombinant proteins**^b^		

PBS (phosphate-buffered saline) tablets	Thermo Fisher Scientific	003002
20× SSC	Thermo Fisher Scientific	15557044
16% Paraformaldehyde (formaldehyde) aqueous solution	Electron Microscopy Sciences	RT15700 (larger sizes also available)
Normal Goat Serum Blocking Solution	Vector Laboratories	S-1000-20
Triton™ X-100	Sigma-Aldrich	X100
VECTASHIELD® PLUS Antifade Mounting Medium	Vector Laboratories	H-1900

**Critical commercial assays**^d^		

RNAscope® Multiplex Fluorescent Reagent Kit v2	ACD	323100-USM
RNAscope® Probe-Mm-Lrrc52	ACD	548201
TSA Plus Fluorescein	Akoya Bioscience	SKU NEL74100 1KT

**Experimental models: organisms/strains**		

C57BL/6J Mice^c^	The Jackson Laboratory	Stock No: 000664
Thy1-YFP-16 transgenic mice (Feng et al., 2000)	The Jackson Laboratory	Stock No: 003709

**Software and algorithms**		

Adobe Photoshop	www.adobe.com	N/A
ImageJ	rsbweb.nih.gov/ij	N/A
Mapping Cochlear Length to Cochlear Frequency (ImageJ plugin)	www.masseyeandear.org/research/otolaryngology/eaton-peabody-laboratories/histology-core	N/A
Imaris	imaris.oxinst.com	N/A
Microsoft Excel	Microsoft.com	N/A
RStudio	rstudio.com	N/A

**Deposited data**		

Calculation of Euclidean distances between sets of immunopuncta (custom code for Rstudio)	https://github.com/thepyottlab/euclidean-distances	N/A
Pillar-modiolar classification and comparison of immunolabeled inner hair cell afferent synapses (custom code for RStudio)	https://github.com/thepyottlab/pillar-modiolar	N/A

**Other**		

iEAR: Inner Ear Antibody Resource(primary antibody inventory)	https://www.thepyottlab.com/resources/	N/A
Image processing using Imaris (complete instruction guide)	https://www.thepyottlab.com/resources/	N/A
Calculation of immunopuncta sizes from z-projections using ImageJ (complete instruction guide)	https://www.thepyottlab.com/resources/	N/A
Dumont #5 fine forceps	https://www.finescience.com/	11254-20
Dumont #55 fine forceps	https://www.finescience.com/	11255-20
Surgical scissors	https://www.finescience.com/	14002-12
Double-Ended Micro-Tapered Stainless Steel Spatula	https://www.fishersci.com/	21-401-10
HybEZ™ II Hybridization System	https://acdbio.com/	N/A (ordered directly from vendor)
Leica SP8 confocal microscope	https://www.leica-microsystems.com/	N/A (ordered directly from vendor)

a Various antibodies can be used depending on the experimental design.

b Alternatives: We have had the most success with the chemicals provided by the commercial vendors indicated. However, chemicals from other vendors may be suitable.

c Aternatives: The strain/line of mouse will depend on the experimental design. No changes in the protocol described here are necessary when using a different strain/line. Moreover, this protocol has been successfully applied to other rodents, including rats (e.g., McLean et al., 2009) as well as gerbils and various mole rats (Barone et al., 2019, Pyott et al., 2020).

d Various probes can be used depending on the experimental design.

## Materials and equipment

**Table:** Phosphate Buffered Saline with Tween-20 (PBT20)

Reagent	Final concentration	Amount
10× PBS	1×	4 mL
10% Tween-20	0.1%	0.4 mL
^ddH^_2_^O^	n/a	35.6 mL
**Total**	**n/a**	**40 mL**

Store between 20°C–22°C for up to 2 years.

**Table:** Methanol (MeOH) with PBT20

Reagent	Final concentration	Amount
Methanol	75%	7.5 mL
PBT-Tween20	n/a	2.5 mL
**Total**	**n/a**	**10 mL**
Methanol	50%	5 mL
PBT-Tween20	n/a	5 mL
**Total**	**n/a**	**10 mL**

Store at 4°C for up to 2 years.

**Table:** Saline sodium citrate buffer (SSC)

Reagent	Final concentration	Amount
20× SSC	0.2×	400 μL
^ddH^_2_^O^	n/a	39.6 mL
**Total**	**n/a**	**40 mL**

Store between 20°C–22°C for up to 2 years.

**Table:** 4% (or 1%) Paraformaldehyde (PFA) in Phosphate Buffered Saline (PBS) (PFA/PBS)

Reagent	Final concentration	Amount
16% PFA	4% (1%)	10 mL (2.5 mL)
10× PBS	1×	4 mL
^ddH^_2_^O^	n/a	26 mL (33.5 mL)
**Total**	**n/a**	**40 mL**

**CRITICAL:** PFA is hazardous and should be handled with gloves and adequate ventilation as outlined in the Material Safety Data Sheet. Proper disposal of solutions containing PFA according to institutional guidelines is required. Store at 4°C and use within one month.

**Table:** Blocking Buffer^a^

Reagent	Final concentration	Amount
5% Normal Goat Serum^b^	5%	2.5 mL
20% (v/v) TX-100	4%	10 mL
10% (w/v) Saponin	0.5%	2.5 mL
10× PBS	1×	5 mL
^ddH^_2_^O^	n/a	30 mL
**Total**	**n/a**	**50 mL**

Store at 4°C and use within one month.

a Syringe filter (0.2 μm) before storing and using.

b Source of serum will depend on the host of the primary and secondary antibodies. For most combinations, normal goat serum is appropriate.

**Table:** Phosphate Buffered Saline with TX-100 (PBT)

Reagent	Final concentration	Amount
10× PBS	1×	5 mL
20% TX-100	0.6%	1.5 mL
^ddH^_2_^O^	n/a	43.5 mL
**Total**	**n/a**	**50 mL**

Store between 20°C–22°C for up to 2 years.

## Step-by-step method details

### Isolation of the inner ear and *in situ* fixation of the organ of Corti

**Timing: 10 min for isolation of the inner ear per animal and between 30 min and 4 h for subsequent fixation.**

This procedure describes isolation of the inner ear from the anesthetized animal for fixation.

***Note:*** This protocol has been successfully used to isolate organs of Corti from mice of both sexes, aged 2 weeks to 2 years.

1. Anesthetize the animal according to the requirements of the institution’s ethical approval. After verifying sufficient depth of anesthesia (by absence of withdrawal reflexes), decapitate the mouse using surgical scissors.
2. Taking care not to damage the temporal bone, bisect the skull and then the palate sagittally using surgical scissors (Figure 1A).

**Figure 1:**
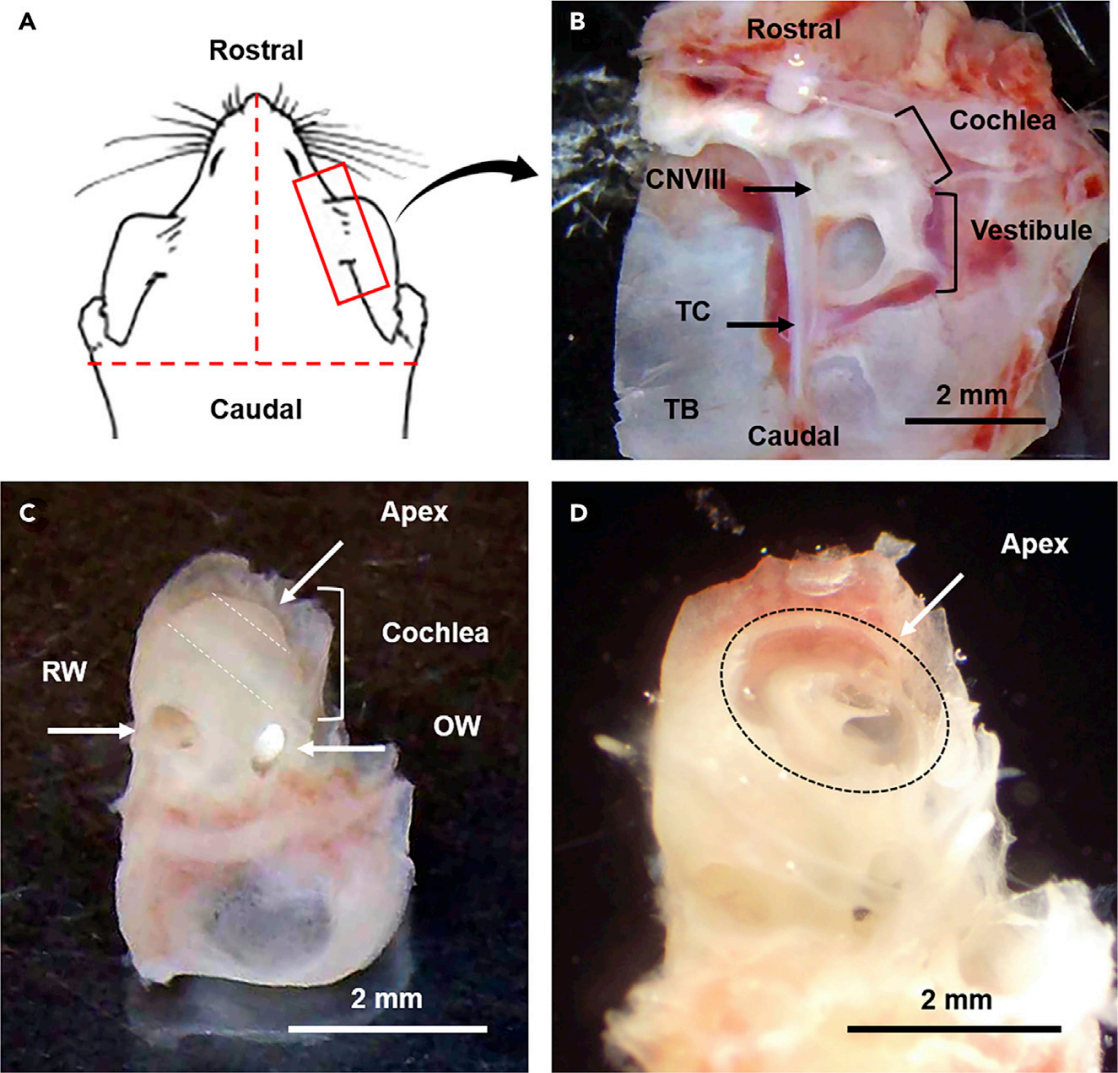
Isolation and preparation of the inner ear for fixation (A) The lines of cut (dashed lines) and region of the temporal bone (red box) containing the inner ear are indicated. (B) The inner ear, comprising the cochlea and vestibule, is attached to the temporal bone (TB) adjacent to the tentorium cerebelli (TC). The auditory and vestibular nerves exit the cochlea and vestibule, respectively, and fuse to form the vestibulocochlear nerve (CNVIII). (C) The cochlea of the isolated inner (now rotated 180°around the vertical axis in the orientation shown in A) shows several anatomical features, including the round window (RW) and oval window (OW). The apex of the cochlea (containing the portion of the organ of Corti, or auditory sensory epithelium, responsive to the lowest frequencies of sound) is marked. The mouse cochlea contains an apical, middle, and basal turn, distinguished by the two dashed lines. (D) A small hole in the bone overlaying the apex has been made to allow better access to fixative and is outlined with a dashed oval. The dissection is shown from a mouse aged 6 weeks old.
3. Remove the brain with surgical scissors or spatula to expose the inner ear embedded in each (left and right) temporal bone.
4. Using the surgical scissors, remove the sections of temporal bone containing the inner ear (easily identified by the semicircular canals) and transfer to ice-cold PBS (Figure 1B).

***Alternatives:*** Temporal bones can be dissected into ice-cold 4% PFA instead of ice-cold PBS to begin fixation more quickly. Temporal bones can also be dissected instead into 0.4% PFA at 20°C–22°C for RNAscope. When dissecting in PFA, be sure to work in a fume hood with adequate ventilation. For electrophysiology or bulk RNA sequencing, dissection of organs of Corti should be performed, respectively, in the appropriate external solution (see methodology in Goutman and Pyott, 2016) or preferred RNA preservation solution (see methodology in Reijntjes et al., 2019).

***Note:*** We use 5-cm plastic petri dishes for dissections.

5. Working under a dissecting microscope, position one pair of forceps around the inner ear (at the junction between the cochlea and semicircular canals) to gently but firmly hold the temporal bone against the bottom of the dissecting dish. The forceps should be pressed down against the temporal bone and dish, rather than squeezed against the inner ear, to secure and prevent damage to the inner ear.
6. Use blunt forceps to remove the inner ear from the temporal bone and auditory bulla.
7. Transfer the isolated inner ear to a new 5-cm dissecting dish containing fresh ice-cold PBS, ice-cold or room temperature 4% or 0.4% PFA, external solution or RNA preservation solution (Figure 1C).
8. Repeat steps 5 and 6 so that both isolated inner ears are in the same dissecting dish.
9. Position one pair of forceps around the inner ear (at the junction between the cochlea and semicircular canals) to gently but firmly hold the inner ear against the bottom of the dissecting dish. The oval and round windows and cochlear turns should be visible (Figure 1C).
10. Using one blade of the forceps, gently scrape and then chip a small hole in the bone covering the apical turn of the cochlea. This hole should be large enough to expose the apical turn. Be careful not to damage the underlying lateral wall or organ of Corti, which should remain covered by the intact lateral wall (Figure 1D).
11. Repeat steps 8 and 9 for the other inner ear.
12. Transfer both inner ears to ice-cold 4% PFA. Fix inner ears for 30 min to 3 h depending on the primary antibodies to be used.

***Note:*** We use 5- or 15-mL plastic centrifuge tubes for fixation.

***Note:*** This methodology can be adapted for fixation of the vestibular sensory epithelia, including the utricle (the utricular macula) and the horizontal and anterior canals (the semicircular canal cristae) instead of or in addition to the organ of Corti. In this case, use blunt forceps to carefully remove the bone covering the roof of the utricle. Use fine forceps to gently open the membranous labyrinth covering the utricular macula and then remove the overlying otoconia. The vestibular epithelia are then isolated after fixation.

**CRITICAL:** In our experience, the duration of fixation (both over- and under-fixation) critically impacts immunoreactivity. We routinely begin with 1 h fixation times but will adjust (up or down) during optimization for a particular antibody. In our experience, fixation for 1 h at room temperature followed by 15 min on ice is best for RNAscope. For electrophysiology (see methodology in Goutman and Pyott, 2016) or bulk RNA sequencing (see methodology in Reijntjes et al., 2019), fixation should not be performed and dissection should proceed immediately to isolation of the (unfixed) organ of Corti.

### Isolation of the organ of Corti

**Timing: 10 min for isolation of the organ of Corti per inner ear**

This procedure describes isolation of the organ of Corti from the inner ear.

13. After fixation (unless an alternative procedure is utilized), transfer the inner ears into a dissecting dish with fresh, ice-cold PBS (or appropriate solution).
14. Again, position one pair of forceps around the inner ear (at the junction between the cochlea and semicircular canals) to gently but firmly hold the inner ear against the bottom of the dissecting dish.
15. Use another pair of forceps to continue removing the bone covering the organ of Corti, beginning with the hole made in the apex and gradually expanding it so that all turns and the most basal hook of the organ of Corti are exposed.
16. Use a pair of fine forceps to “peel” the lateral wall away from organ of Corti. Begin by pinching the lateral wall at the apical turns (where it is more loosely associated with the organ of Corti) and gradually remove it from the organ of Corti using an unwinding motion.

**CRITICAL:** Take care not to damage the organ of Corti by carefully positioning the forceps between the lateral wall and the bone. Removing the bone without damaging the underlying organ of Corti and removing the lateral wall without disrupting the organ of Corti becomes more difficult in basal compared to apical turns and in older (> 6 weeks) compared to younger animals. Nevertheless, in our experience, dissections from animals older than 6 weeks (and up to two years of age) are comparable and quite doable with fine forceps and some practice. (See also problem 1 below).

17. Once the entire organ of Corti has been exposed, insert one blade of the fine forceps underneath the basal hook and gently push up to detach the basal turn (with some underlying bone) from the modiolus. Work slowly.
18. Once the most basal turn is loose, insert one blade of the fine forceps between the apical turn of the organ of Corti and the modiolus and gently push up from the underside of the organ of Corti to detach it from the modiolus. Again, work slowly.
19. Continue in this way to work around the turns of the organ of Corti to release the entire organ of Corti from the modiolus.

***Note:*** In some cases, it may be necessary to use a small dissecting knife or microscissors to cut the central projections of the spiral ganglion cells to release the organ of Corti from the modiolus.

***Note:*** In some cases, the organ of Corti may need to be removed in two (or more) pieces rather than one piece. Since the immunostained organ of Corti will later need to be cut for mounting on slides, isolation in multiple pieces is not necessarily a problem although it does make the solution exchanges required for immunostaining more difficult. (See also problem 2 below.)

***Note:*** If desired, the vestibular sensory epithelia can now be isolated (see methodology in Sadeghi et al., 2014, Schuth et al., 2014, Greguske et al., 2021).

**Figure 2:**
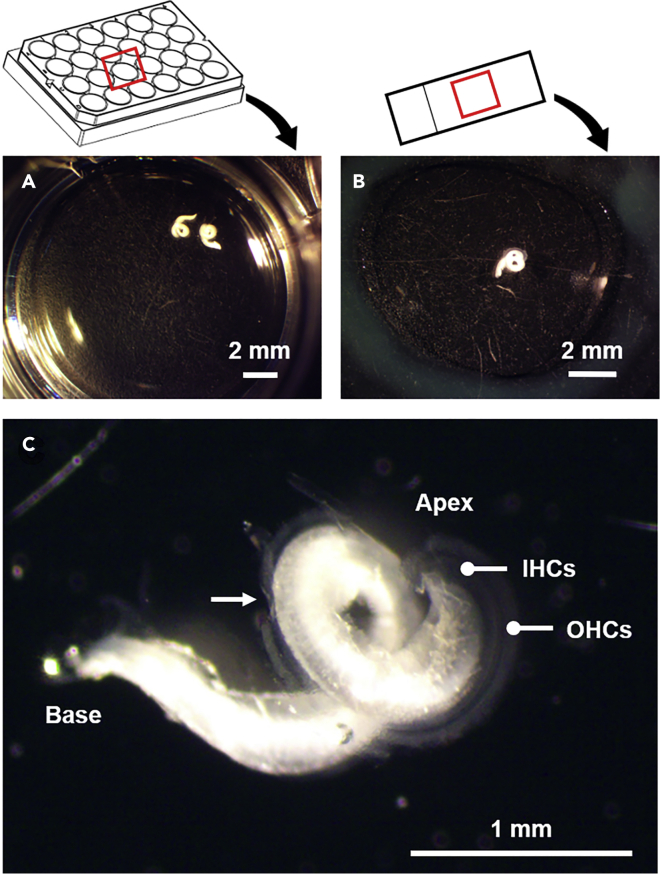
Fixed and isolated organs of Corti for RNAscope and immunostaining Isolated organs of Corti are processed in 24-well plates filed with solution (A) or on slides in solution contained within a circle-shaped hydrophobic barrier (B). (C) The organ of Corti spirals from the apex to base. Even at low magnification, the orderly arrangement of the inner hair cells (IHCs) and outer hair cells (OHCs) in rows spiraling the length of the organ of Corti is visible. The arrow marks a section of the organ of Corti that has been damaged during dissection.

***Note:*** We use 24-well (cell culture) plates for RNAscope and immunostaining (Figure 2A). Each well is filled with 300 to 400 μL of solution. As required for the specific experiment, we put one to four organs of Corti into a single well. Solutions are exchanged using a (1000 μL) micropipettor while samples remain in the same well. Care needs to be taken so as not to pipette the samples together with the solution. Fewer organs of Corti per well makes the solution exchanges required for immunostaining easier and may be required when keeping track of individuals and/or specific genotypes. On the other hand, more organs of Corti per well reduces the amount of solution necessary (which is advantageous when working with expensive reagents). Alternatively, wells can be pre-filled with the required solution and samples can be moved between wells. Samples should be transferred gently between wells using fine forceps. For RNAscope assays we also use microscope slides (Figure 2B). Organs of Corti are placed in solution within a circle-shaped hydrophobic barrier (see step 28 “Fluorescence in situ hybridization in the organ of Corti with RNAscope” below).

***Note:*** For electrophysiology (see methodology in Goutman and Pyott, 2016) or bulk RNA sequencing (see methodology in Reijntjes et al., 2019), the isolated organs of Corti should be processed immediately.

20. Once the organ of Corti has been isolated, use a pair of fine forceps to “peel” the tectorial membrane away from the organ of Corti. Use one pair of fine forceps to gently hold the organ of Corti. Take a second pair of fine forceps to pinch the tectorial membrane at the apical turn (where it is more loosely associated with the organ of Corti) and gradually remove it from the organ of Corti using an unwinding motion.
21. Remove any excess bone left attached to the organ of Corti and transfer the organ(s) of Corti to a 24-well (cell culture) plate for either RNAscope or immunostaining (Figure 2).

### Fluorescence *in situ* hybridization in the organ of Corti with RNAscope

**Timing: 2 h on the first day and 6–7 h on the second day**

This procedure describes single molecule *in situ* hybridization with RNAscope in the isolated and fixed organ of Corti. The described protocol is adapted from Kersigo et al. (2018) and the RNAscope Multiplex Fluorescent Reagent Kit v2 User Manual (https://acdbio.com/system/files_force/USM-323100%20Multiplex%20Fluorescent%20v2%20User%20Manual_10282019_0.pdf?download=1). Steps 22**–**33 are completed on day 1 and steps 34**–**51 are completed on day 2.

22. Turn on HybEZ™ hybridization oven. The hybridization oven will automatically load the RNAscope protocol and adjust the temperature to 40°C in about 10 min.
23. Prepare target probe in hybridization solution. Target probes come in different stock concentrations depending on the channel assigned to them. C1 probes can be directly aliquoted and warmed. C2 and C3 probes need to be diluted in probe diluent that has been pre-warmed to 40°C in the hybridization oven for 10 min and then cooled to room temperature. The C2 or C3 probes should be briefly centrifuged before being mixed with probe diluent.

***Note:*** We typically dilute 2 μL of either C2 or C3 probe in 100 μL of probe diluent in either 0.5- or 1.5-mL centrifuge tubes. Here we describe detection of transcripts with a single probe because we often combine RNAscope with subsequent immunofluorescence. However, the protocol is similar for the simultaneous detection of multiple transcripts using C1, C2, and C3 probes.

24. After fixation in 4% PFA (step 21 from “Isolation of the organ of Corti” above), wash samples (organs of Corti) in PBT20 in a 24-well plate (see Note in step 21 and Figure 2B) for 3 times 10 min each at room temperature.
25. Dehydrate samples in a 24-well plate through increasing MeOH/PBT20 solution gradients: 50%, 75%, and 100% MeOH; each step should last 5 min and be performed at room temperature.
26. Rehydrate samples in a 24-well plate through decreasing MeOH/PBT20 solution gradients: 75%, 50%, 0% MeOH; again, each step should last 5 min and be performed at room temperature.

***Note:*** Samples can be stored in 100% MeOH (after step 25) at −20°C for at least 3 weeks. In our experience, dehydration followed by rehydration improves probe permeabilization and is, therefore, desirable even if samples are not stored at −20°C before further processing.

27. Wash samples in PBT20 in a 24-well plate 3 times 5 min each at room temperature.
28. During the last PBT20 wash, prepare slides for proteinase incubation by drawing a circle-shaped hydrophobic barrier with a PAP-pen (Figure 2B). Place approximately 3 drops (50**–**75 μL) of Proteinase III inside the hydrophobic circle on the slide.
29. Transfer samples from the 24-well plate (step 26) to the Proteinase III solution (step 28) and incubate for 8 min on the slide at room temperature.
30. Stop protein digestion by moving samples back to a 24-well plate containing PBT20 and wash samples in PBT20 3 times 5 min each at room temperature.
31. While samples are being washed in PBT20, line the bottom of humidity control tray of the hybridization oven with paper towels moistened with distilled water.
32. Prepare slides for hybridization: make a circle-shaped hydrophobic barrier on slides using a PAP-pen; place the slides on the oven’s slide rack; position the slides on the rack in the humidity control tray; and pipette approximately 50**–**75 μL of the target probe hybridization solution (from step 23) into the center of each hydrophobic circle.

***Note:*** During incubation in the hybridization oven (15**–**18 h), the hybridization solution will flatten and expand considerably. Leave space between solution and hydrophobic barrier to avoid leakage across the barrier. (See also problem 3 below.) To avoid cross contamination, we typically use one slide per hydrophobic barrier. In our experience, the type of slides used for hybridization is not critical.

33. Carefully transfer samples from the 24-well plate with PBT20 (step 30) onto slides with hybridization solution (step 32). Place the humidity control tray with slide rack into hybridization oven and incubate samples at 40°C for 15**–**18 h.
34. Carefully remove the tray with slide rack from the hybridization oven and transfer samples to a 24-well plate with 0.2× SSC. Wash in 0.2× SSC 3 times 15 min each.
35. While samples are being washed in 0.2× SSC, aliquot preamplifier hybridization solution (Amp1), signal enhancement solution (Amp2), amplifier hybridization solution (Amp3), horseradish peroxidase (HRP) and HRP-blocker and equilibrate to room temperature before using.

***Note:*** We typically warm these solutions in 1.5-mL centrifuge tubes. These solutions are provided in dropper bottles, with 3 drops being approximately 75 μL, the amount needed per sample for subsequent hybridization reactions.

36. Refix samples briefly: pipette 75 μL of 4% PFA into a circle-shaped hydrophobic barrier on a slide; transfer samples (from step 34) into the PFA; and fix for 10 min at room temperature.
37. Wash samples in 0.2× SSC in a 24-well plate 3 times 5 min each at room temperature.
38. Incubate samples in preamplifier hybridization solution (Amp1) in a circle-shaped hydrophobic barrier on a slide for 35 min at 40°C in the hybridization oven.
39. Wash samples in 0.2× SSC in a 24-well plate 3 times 15 min each at room temperature.
40. Incubate samples in signal enhancement solution (Amp2) in a circle-shaped hydrophobic barrier on a slide for 20 min at 40°C in the hybridization oven.
41. Wash samples in 0.2× SSC in a 24-well plate 3 times 15 min each at room temperature.
42. Incubate samples in amplifier hybridization solution (Amp3) in a circle-shaped hydrophobic barrier on a slide for 35 min at 40°C in hybridization oven.
43. Wash samples in 0.2× SSC in a 24-well plate 3 times 15 min each at room temperature.
44. Incubate tissue in horseradish peroxidase (HRP) in a circle-shaped hydrophobic barrier on a slide for 15 min at 40°C in hybridization oven. Choose the appropriate HRP corresponding to the channel on which the target probe is detected.
45. While samples are incubating in HRP, prepare fluorescein: dilute 1 μL TSA® Plus fluorescein in 1500 μL TSA buffer.

***Note:*** Approximately 75 μL diluted fluorescein is needed per sample. In our experience, extra diluted fluorescein can be stored light shielded at 4°C for at least one month.

***Alternatives:*** Instead of fluorescein, TSA® Plus cyanine 3 (Cy3), cyanine 5 (Cy5), or Opal™ dyes from Akoya Biosciences can be used (see key resources table). If choosing an alternative fluorophore, dilution may need to be adjusted based on signal intensity. Be careful to choose fluorophores that are mutually compatible between targets and subsequent immunofluorescence.

46. Wash samples in 0.2× SSC in a 24-well plate 2 times 2 min each at room temperature.
47. Incubate tissue in TSA Plus® fluorescein for 30 min at 40°C in the hybridization oven.
48. Wash samples in 0.2× SSC in a 24-well plate 2 times 2 min each at room temperature.
49. Stop horseradish peroxidase reaction by incubating samples in HRP blocker in a circle-shaped hydrophobic barrier on a slide for 15 min at 40°C in the hybridization oven.
50. Wash samples in 0.2× SSC in a 24-well plate 2 times 2 min each at room temperature.
51. Wash samples in 1× PBS in a 24-well plate 2 times 2 min each at room temperature.

***Note:*** Samples can proceed directly to immunofluorescent staining (see “immunofluorescent staining of fixed organs of Corti” below) or be immediately mounted (see “mounting of prepared organs of Corti” further below). Alternatively, samples can be stored in 1× PBS before subsequent processing. If pausing before continuing immunofluorescent staining, we recommend storing samples in blocking buffer (see Note in step 55 below).

### Immunofluorescent staining of fixed organs of Corti

**Timing: 8 h to 2 days (with approximately 2 h of active time)**

This procedure describes immunofluorescent staining of the isolated and fixed organs of Corti.

52. Block (and permeabilize) samples in the blocking buffer for at least 1 h at room temperature.

**Pause point:** Once the isolated organs of Corti are placed in blocking buffer, the protocol can be paused. Reasons for pausing may include waiting for the outcome of genotyping results so that a subset of samples can be identified for immunostaining or to collect organs of Corti for a single round of immunostaining. Isolated organs of Corti (as well as isolated vestibular epithelia) will store without loss of immunoreactivity for days to weeks (and perhaps longer) in blocking buffer at 4°C. When storing, it is important to make sure that the organs of Corti remain submerged in blocking buffer. We often use an excess of blocking buffer (500 to 1000 μL per well in a 24-well plate) and then seal with parafilm.

53. Use a (1000 μL) micropipettor to remove the blocking buffer (see Note in step 21 above).
54. Add the primary antibody solution diluted to the appropriate concentration in blocking buffer.
55. Incubate organs of Corti in the primary antibody solution at least 4 h or up to 48 h at room temperature. See also problem 5 below.

***Note:*** Many laboratories routinely perform incubations at 4° C. In our experience immunoreactivity is improved when incubating at room temperature (even for 48 h incubations).

***Note:*** We have compiled an inventory of primary antibodies (iEAR: Inner Ear Antibody Resource) for immunostaining the isolated organ of Corti (and vestibular sensory epithelia) from the inner ear. The link to this inventory is provided in the key resources table above. (See also problem 4 below.)

56. Rinse the samples with PBT 3 times for 10 min each at room temperature.

**Pause point*:*** Samples can be rinsed longer or even stored in PBT at 4° C (in the refrigerator) if necessary or convenient.

57. Remove PBT solution and add the secondary antibody solution diluted to the appropriate concentration in blocking buffer.
58. Incubate organs of Corti in the secondary antibody solution at least 4 h or up to 24 h at room temperature.

***Note:*** Vestibular sensory epithelia are immunostained using the same protocol (see methodology in Sadeghi et al., 2014, Schuth et al., 2014, Greguske et al., 2021).

59. After incubating in the secondary antibody, rinse the samples with PBT 3 times 10 min each and once with PBS at room temperature.

### Mounting of prepared organs of Corti

**Timing: 5 min per organ of Corti**

This procedure describes mounting of immunofluorescent stained organs of Corti on glass microscope slides.

**Figure 3:**
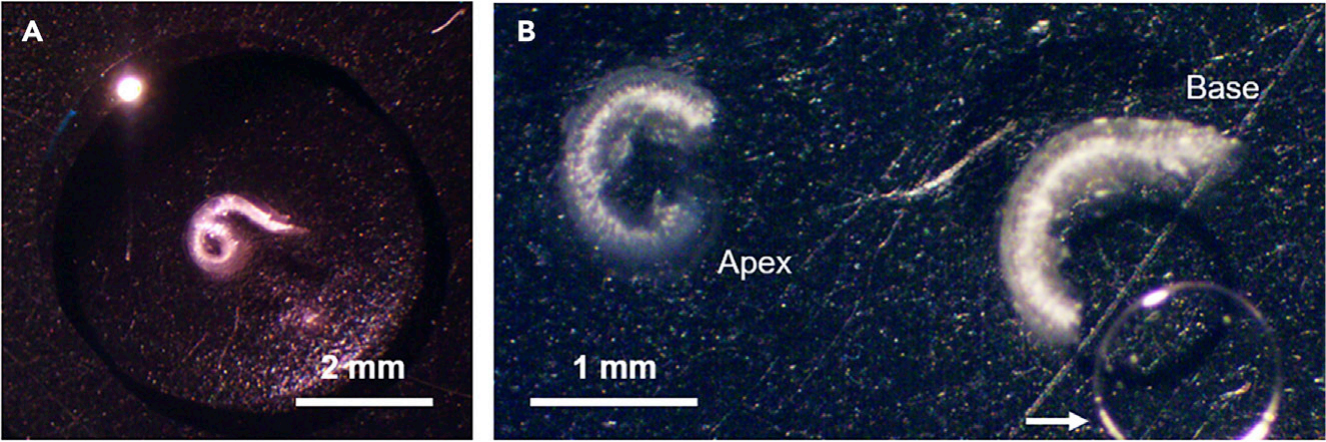
Mounting organs of Corti for microscopy (A) Organs of Corti are placed into individual drops of mounting medium. (B) To prevent turns of the organ of Corti from overlapping, the organ of Corti is cut into two or three pieces before mounting with a glass coverslip (as shown here). The apex and base are indicated. The arrow marks an air bubble caught inadvertently under the coverslip.

60. Place a drop of Vectashield (or similar mounting medium) for each organ of Corti to be mounted on a clean microscope slide.
61. Use fine forceps to transfer the piece(s) of organ of Corti into the drop of mounting medium. To minimize damage to the organs of Corti, use a lifting rather than pinching motion to transfer the samples. Under a dissecting microscope, use the forceps to gently position the pieces so that the basilar membrane is facing the slide and the organ of Corti is facing the coverslip. Segments of organ of Corti are ideally positioned close to each other to make subsequent imaging more convenient (Figure 3A).

**CRITICAL:** Various features indicate the proper orientation of the organs of Corti when mounting. Most notably, when the organ of Corti is intact, the apical end spirals upward and away from the slide when the basilar membrane side is appropriately facing the slide. The organs of Corti will flatten once mounted. Less notable, there is a ridge of tissue where the tectorial membrane attaches. The presence of this ridge identifies the organ of Corti side.

62. Carefully overlay a glass coverslip and use nail polish to adhere the coverslip to the slide (Figure 3B).
63. Label slides appropriately.

**CRITICAL:** In our experience, organs of Corti are easier to mount (and less likely to become folded) when they are cold. Therefore, if organs of Corti are at room temperature, we recommend allowing the organs of Corti to first cool to 4° C. (See also problem 5 below.)

***Note:*** We typically mount four organs of Corti on a single microscope slide.

**CRITICAL:** Careful mounting of the samples will better preserve their 3D structure and improve subsequent quantitative image analyses. (See also problem 5 below.)

***Note:*** Vestibular sensory epithelia are mounted using the same protocol (see methodology in Sadeghi et al., 2014, Schuth et al., 2014, Greguske et al., 2021).

**Pause point:** In our experience, mounted slides can be stored in the dark at 4°C for at least one year.

### Overlaying cochlear frequency maps

**Timing: 15 min per organ of Corti**

This procedure describes overlaying of tonotopic maps on mounted organs of Corti. If identification of precise tonotopic regions is not necessary, this procedure can be omitted.

**Figure 4:**
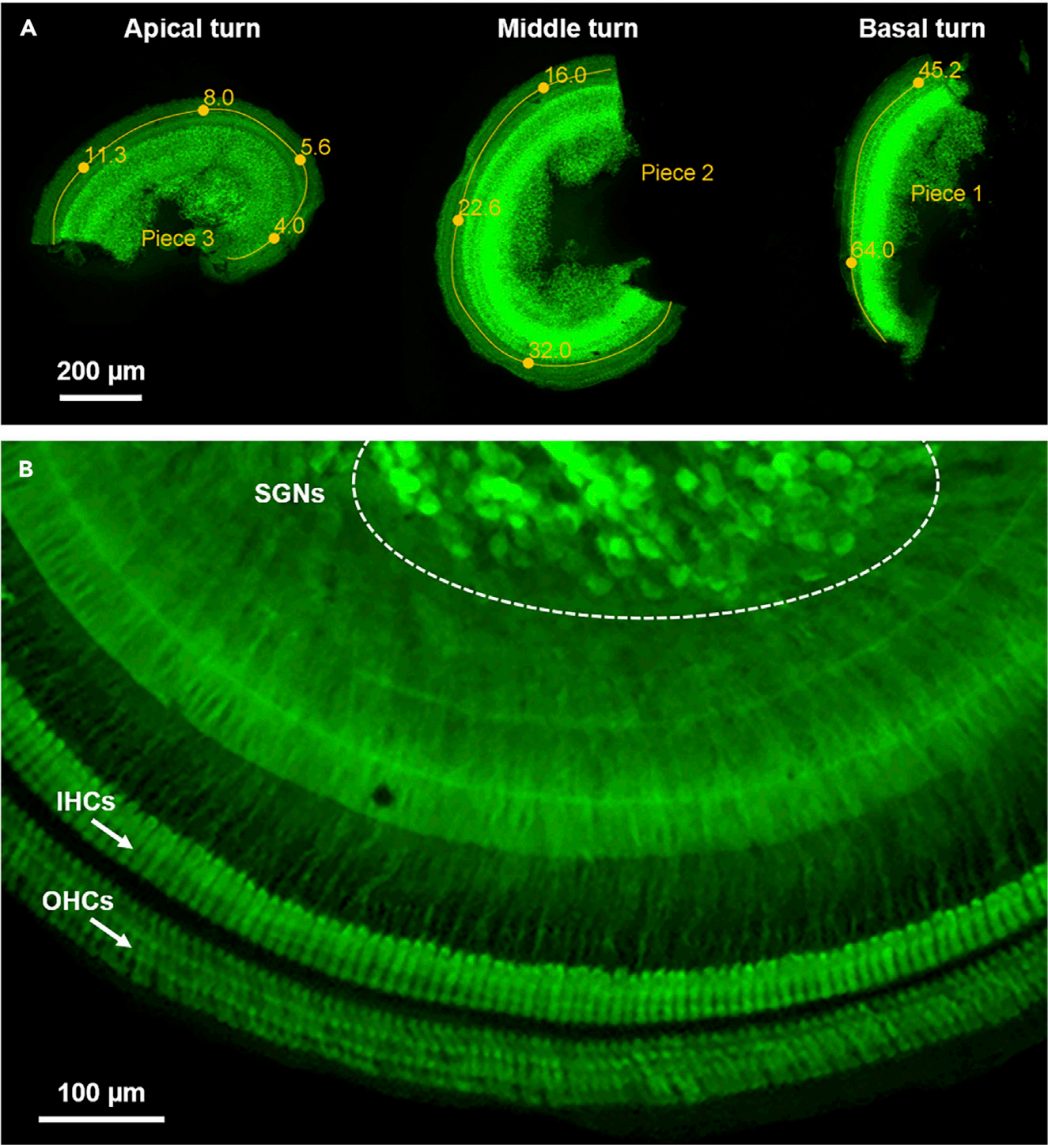
Epifluorescent imaging of the immunostained and mounted organ of Corti (A) Three lower magnification epifluorescent micrographs of the apical, middle, and basal turns of the immunostained and mounted organ of Corti have been combined into a single digital image to overlay a tonotopic map (yellow). (B) A higher magnification epifluorescent micrograph reveals the spiral ganglion neurons (SGNs, circled) and rows of inner hair cells (IHCs) and outer hair cells (OHCs). In both A and B, the organ of Corti is immunostained with rabbit anti-calretinin and goat anti-Rabbit (488).

***Note:*** If multiple images are taken, it may be necessary to montage images of a single segment and/or combine separate images of different segments into a single image file (Figure 4B).

64. Obtain low magnification (5× epifluoresecent) micrographs of the entire organ of Corti (Figure 4A). Often multiple images will need to be taken to capture the entire organ of Corti.

65. To montage images in Adobe Photoshop:
  a. In Photoshop, open images to montage by going to File → Script → Load files into stack and then selecting the images to montage.
  b. In the layers panel, select all layers (to be montaged) and then go to Edit → Auto Aligns Layers → Reposition.
  c. Flatten layers by going to Layer → Flatten Image.
66. To combine separate images into a single image file:
  a. In Photoshop, open one of the images (or keep the montaged image from the previous step 65c open).
  b. Make the canvas size larger (to accommodate placement of the new images) by going to Image → Canvas Size. (Keeping the anchor to left or right when the canvas is enlarged is best and makes space on either the right or left for placement of the new images).
  c. Open the image to be added in a new window.
  d. Use the rectangular marquee tool to outline the region of interest containing the segment to be added and then copy (Ctrl-C) the selected segment.
  e. In the original image, paste (Ctrl-V) the copied segment.
  f. If necessary, reposition the copied segment using the Move Tool.
  g. Once all segments have been incorporated into a single file, flatten layers by going to Layer → Flatten Image.
  h. If necessary, crop extra canvas using the crop tool.
  i. Save in RGB format as [filename]_merged.tif (or whichever name/format is preferred).

***Note:*** We use tools developed by researchers at the Eaton-Peabody Laboratories to overlay cochlear frequency maps on low magnification images of the organs of Corti (see “Mapping Cochlear Length to Cochlear Frequency” in the key resources table). A summary of these instructions is included here for completeness.

67. To add the cochlear place-frequency maps using ImageJ:
  a. Download ImageJ from NIH.
  b. Download “Measure_Line.class” and add it to the directory ImageJ\Plugins\Tools.
  c. In ImageJ, open the montaged/combined image (saved in RGB format in step 66i).
  d. From the main toolbar, go to Plugins → Tools → Measure Line.
  e. From the main toolbar, select the “segmented” line tool.
  f. Mark the length of each segment of organ of Corti from base to apex: single click to begin, single click to insert vertex within a segment, and double click to end marking segment”; click “d” after each segment has been marked.
  g. Once all segments are marked, click “a” to annotate for the selected species.
  h. To identify specific tonotopic regions, click “p” for point and select the “hand” tool from the main toolbar. The exact frequency is indicated in the main toolbar when dragging the hand across the annotated tonotopic map. Clicking on the tonotopic annotation will annotate the map with the exact frequency at that location.
  i. Save the image with the overlaid frequency map in RGB format as [filename]_mapped.tif (or whichever name/format is preferred).

***Note:*** Steps a and b need to be performed only once, whereas the subsequent steps (notably step d) need to be completed upon opening each new image.

### Obtaining confocal micrographs

**Timing: 15 min to 1 h depending on number of samples scanned and scanning parameters used.**

***Note:*** This procedure describes methodology to obtain single or stacks of confocal micrographs. Scanning parameters will vary between the specimens examined, the microscopes used, and the image analysis required. We, therefore, point out a few general guidelines. Excellent resources are available elsewhere (e.g., Murray, 2013). A summary of these instructions is included here for completeness.

68. Select the appropriate lasers, filters, detection windows and scanning configuration depending on the fluorophores (secondary antibodies) used. (If necessary, verify the appropriate pinhole diameter, generally “1 airy”.)
69. Use the low magnification images with superimposed maps (saved in step 67i from the preceding procedure) to identify the appropriate tonotopic region under high magnification.

***Note:*** We use a 63× oil objective (N.A. 1.2) to obtain high magnification images for quantitative image analyses.

**Figure 5:**
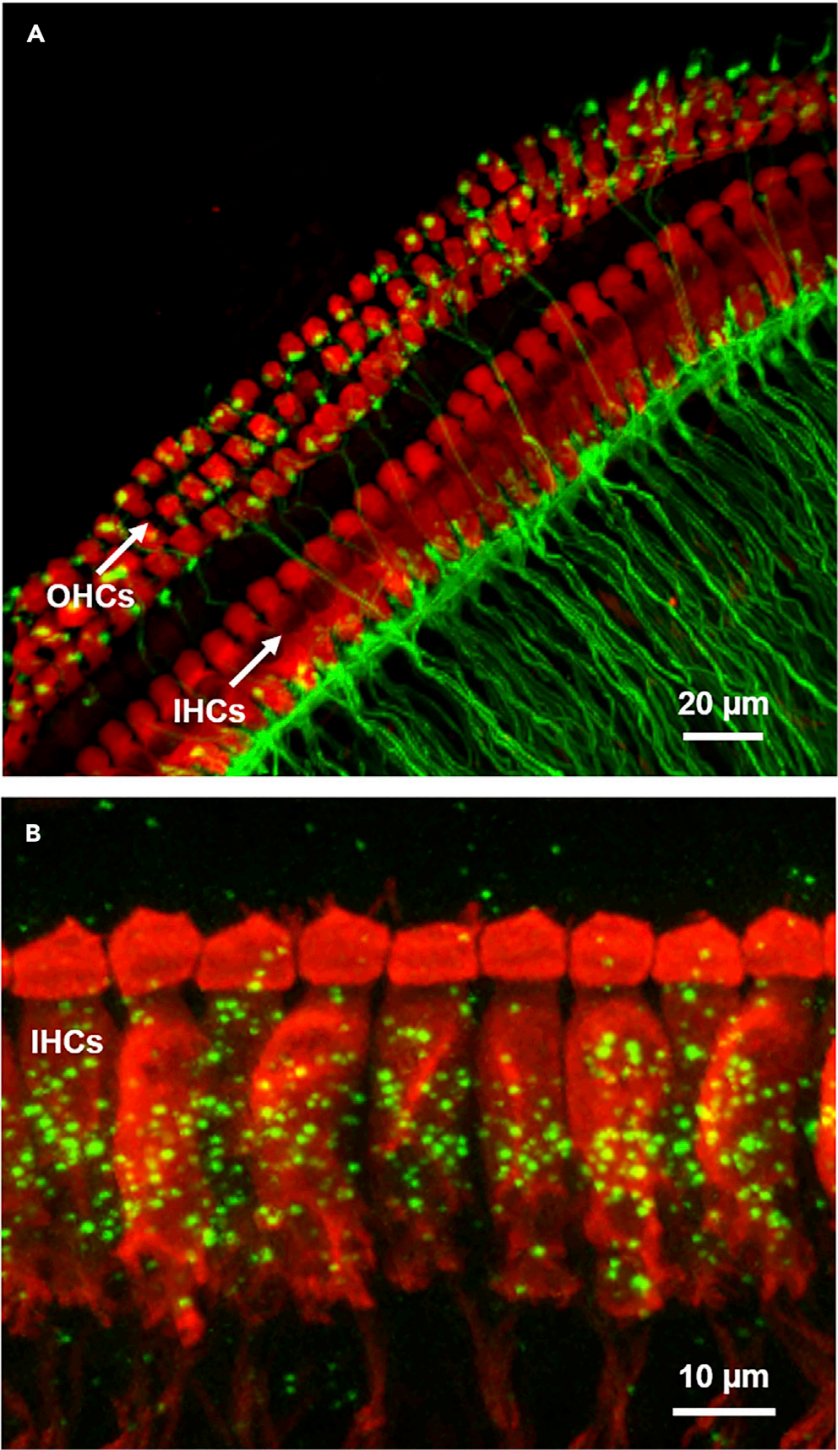
Z-projection through a stack of confocal micrographs of an organ of Corti With identification of the appropriate markers, a variety of structures can be identified in precisely defined tonotopic regions of the immunostained organ of Corti. (A) Organ of Corti isolated from Thy1-YFP-16 transgenic mice (Feng et al., 2000) express yellow fluorescent protein (green) in the myelinated neurons contacting the inner hair cells (IHCs) and outer hair cells (OHCs). IHCs and OHCs are immunostained with rabbit anti-MYO6 (red). A video of a 3D rotation is available as a supplemental video (Methods video S1). (B) Organ of Corti showing abundant expression of Lrrc52 transcript (green) detected by RNAscope in the inner hair cells (IHCs). IHCs are immunostained with rabbit anti-calretinin (red).

***Note:*** Scanning protocols will vary depending on the subsequent image analysis. The optimal z-step size can often be calculated by the acquisition software. Generally, our stacks consist of about 90 scans (z step size = 0.3 μm) collected frame by frame (for sequential scanning) with a scan speed of 100 Hz (lines per second) and resolution of 1024 X 1024. These parameters are suitable for the quantitative image analyses described below.

***Note:*** Deconvolution of confocal images can be used to “deblur” images, which can be helpful with subsequent image analysis. The details for performing deconvolution will vary with the software and algorithms used and should be considered carefully before applying. Excellent resources are available elsewhere (e.g., Swedlow, 2013).

70. Once the appropriate region of interest has been identified, set the laser intensity, gain, and offset so that the brightest parts of the image are just saturated (generally appearing “blue” when viewing under the “glow over glow under” color map or LUT).
71. Repeat this step for the other fluorophores, and, if necessary, adjust so that there is no “cross talk” between channels.
72. When obtaining a stack of images, identify the relative top and bottom of the region of interest (for example, all afferent synapses at the bases of the inner hair cells).
73. Collect the required number of stacks at the optimal required resolution. After scanning, viewing projections through the entire stack is useful to verify that the correct region has been scanned (Figure 5).

## Expected outcomes

The described method allows a fast and straightforward approach to perform RNAscope and immunofluorescently label the intact organ of Corti without prolonged fixation, embedding, or decalcification. In this way, transcript and protein localization and expression patterns can be determined along the tonotopic axis, along the hair cell apical-basal and pillar-modiolar axes, as well as relative to one another. The protocol is adapted at indicated steps for other techniques, such as electrophysiology and RNA sequencing.

## Quantification and statistical analysis

This suite of procedures allows quantitative image analysis of 2D and 3D reconstructions of imaged organs of Corti and is summarized below. Some of these require code developed in Rstudio and made freely available by our group (see key resources table).

### Calculation of immunopuncta sizes from z-projections using ImageJ

**Timing: 10–30 min per region of interest**

**Figure 6:**
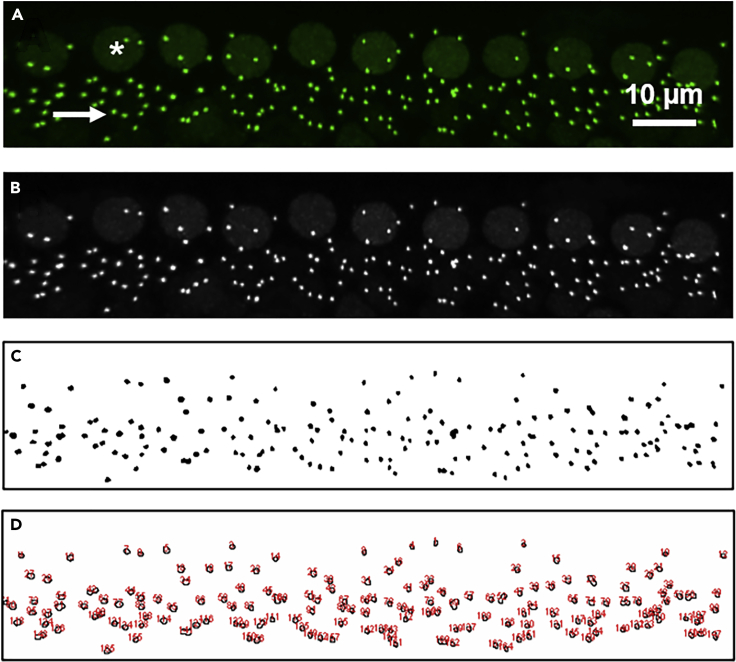
Quantitative 2D image analysis using ImageJ (A) Z-projection through a stack of confocal micrographs of an organ of Corti immunostained with anti-CTBP2 to label IHC nuclei (larger, fainter green immunolabels with example marked by an asterisk) and pre-synaptic ribbons (smaller, brighter green labels immunolabels with example marked by an arrow) bright. (B) Grayscale image of A for subsequent processing. (C) Black and white (inverted) image of B for subsequent processing. (D) Detected puncta with properties (e.g., diameters and surface areas) available for export. Complete instructions are available from the lead contact.

This procedure describes quantification of immunopuncta sizes (e.g., diameters and/or surface areas) from Z-projections in ImageJ (Figure 6) (Pyott et al., 2004, Rohmann et al., 2015, Pyott et al., 2020).

1. In Photoshop, open image (flattened z-projection saved from confocal scanning acquisition software).
2. To rotate and crop to obtain the appropriate region of interest (Figure 6A):
  a. Right click on Eyedropper Tool to obtain the Ruler Tool.
  b. Draw the line of rotation (e.g., along line of inner hair cell nuclei)/
  c. Go to Image → Image Rotation Arbitrary and click OK.
  d. Crop image to exclude regions from analysis.
3. Convert to grayscale image by going to Image → Mode → Grayscale (Figure 6B).
4. If necessary, adjust contrast by going to Image → Auto Contrast.
5. Save as [filename]_ps.tif (or whatever name preferred).
6. In ImageJ, open processed image ([filename]_ps.tif).
7. Set scale by going to Analyze → Set Scale and enter scale (pixels per μm).
8. Convert image to a binary (black and white file) by going to Process → Binary → Make Binary.
9. Save as [filename]_ps_binary (or whichever name/format is preferred).
10. To determine areas using analyze particles (Figures 6C and 6D):
  a. Set “size pixel” based on the approximate size of puncta.
  b. Adjust “circularity” to include/exclude only more/less spherical objects.
  c. Set “show” to “show outlines”.
  d. Set to “display results” and “clear results” (if you want to clear results between analyses).
11. The Results window displays measurements. Measurements displayed can be set by clicking “Results” on the menu.
12. Copy raw data to analysis software (e.g., Excel).
13. If needed, add a scale bar by going to Analyze → Tools → Scale bar.
14. Save as [filename]_ps_outlines (or whatever name preferred).

### Image processing using Imaris

**Timing: varies (but can be estimated at about 1 h per region of interest to complete all steps)**

**Figure 7:**
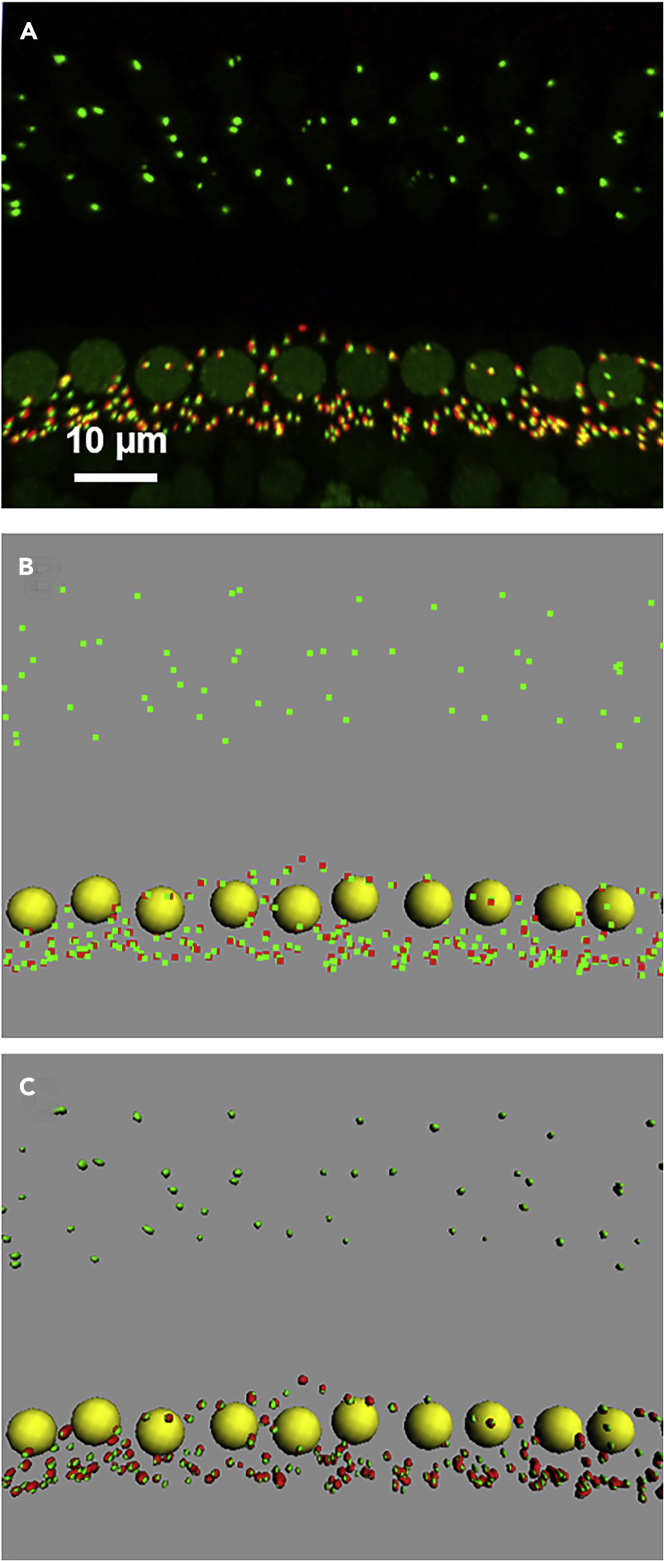
Quantitative 3D image analysis using Imaris and custom script (A) 3D reconstruction of a stack of confocal micrographs of an organ of Corti immunostained with mouse anti-CTBP2 to label IHC nuclei and pre-synaptic ribbons (green) and rabbit anti- GluR2/3 to label postsynaptic glutamate receptor clusters (red). (B) Spots detection of IHC nuclei (yellow spheres), pre-synaptic ribbons (green squares) and post-synaptic glutamate receptor clusters (red squares) for counting and spatial (x, y, z) localization. (C) Surface detection of pre-synaptic ribbons (green surfaces) and post-synaptic glutamate receptor clusters (red square surfaces) for volume analysis and spatial (x, y, z) localization.

**Figure 8:**
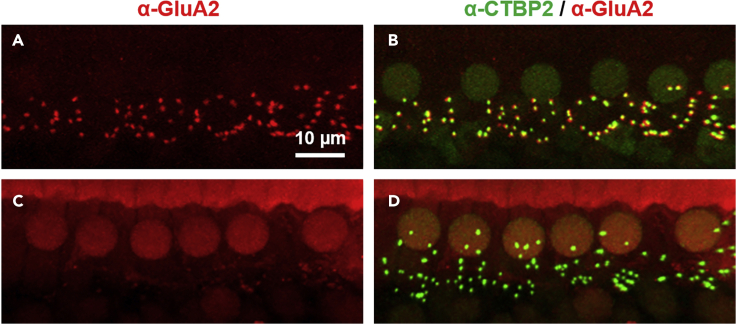
Non-specific immunoreactivity (problem 3) Z-projections through a stack of confocal micrographs of an organ of Corti immunostained with anti-CTBP2 (green) to label presynaptic ribbons and anti-GluR2 (red) to label postsynaptic terminals. In panels (A and B), GluA2 immunoreactivity is clustered (A) and adjacent CTBP2-labeled presynaptic presynaptic ribbons (B). In contrast, in panels (C and D), GluA2 immunoreactivity is diffuse and not clearly clustered to the postsynaptic terminals (C) adjacent the CTBP2-labeled presynaptic ribbons (D).

This set of procedures provides tips for working with Imaris to 1) reconstruct three-dimensional images from stacks of confocal images (Figure 7A); 2) “crop” regions of interest for further analysis; detect immunopuncta (Figure 7B) or fluorescently labeled RNA targets (Figure 5B) with the “spots” function; 3) create surfaces for immunopuncta of interest with the “surfaces” function (Figure 7C); and 4) export values of subsequent analysis (including numbers, volumes, and x, y, z coordinates). This documentation with additional instructional images is provided in the key resources table. 3D renderings in Imaris can be visualized and exported as either projections (e.g., Figures 5, 7, and 8) or animations (e.g., Methods video S1).

15. Tips for opening and examining files in Imaris:
  a. Adjust intensity (lower arrow) and contrast (upper arrow) in the “display adjustment” screen. Go to Edit → Show Display Adjustment (or press Ctrl+D) to show the “display adjustment” screen. Channels can be turned on or off from viewing by selecting or deselecting from this window.
  b. Press the escape key to switch between the selection (pointer) and the rotation (double arrows) mode.
  c. To zoom in or out, scroll in or out.
  d. To rotate, left click and hold.
  e. To drag, right click and hold.
  f. Save files in.ims format (preferably ”[original filename]_Edit”), and save files often!

16. Selecting regions of interest (ROIs) using a “free form” crop in Imaris
  Sometimes it is necessary to analyze only a specific region of interest (ROI; for example, inner hair cells but not outer hair cells or even a subset of hair cells from the entire field of view). While Imaris has a cropping feature, it allows only square ROIs to be cropped. To perform a square crop, go to Edit > Crop 3D. Follow the directions below to crop a free form ROI. In this protocol, cropping is actually done by “masking” everything outside a selected ROI that is defined by a surface.
  a. Add a surface by clicking the Surface function (blue blobs) in the properties window. The surface can be renamed later.
  b. “Skip” automatic creation (wizard).
  c. Go to the Paint Brush → Contour → Mode and select “drawing mode” to insert a line with a vertex every 1 μm. This distance can be adjusted as appropriate. Adjust the slice position slider to view the entire stack.
  d. To demarcate the ROI, toggle to the select mouse pointer (single arrow) by pressing Esc. Then press Ctrl+Space to get the crosshair pointer. Now outline the ROI while holding the left click. Be sure to press Ctrl+Space (to go from the cross hair to single arrow pointer) immediately after selecting the ROI.
  e. Go to the Paint Brush → Contour → Board and hit “Copy”, scroll through to the opposite end of the stack with the slice position slider, hit “Paste,” and then hit “Create Surface.” This will create a surface of the outline through the entire stack.
  f. To “mask” (not see or analyze) the image outside of this ROI (surface), go to Pencil and enter Mask all. Select the channel to be masked. Be sure to deselect the “duplicate channel before masking” option. Repeat masking for all channels to be masked.
  g. Deselect the surface to view the ROI.

17. Detecting immunopuncta of interest with the “spots” function

Methods video S1. Animation of a rotated Z-projection through a stack of confcal micrographs of an immunostained organ of Corti (shown in Figure 5), related to “Image processing using Imaris”, steps 15-21 in the “quantification and statistical analysis” section

Immunopuncta can be marked for counting and determination of x, y, z coordinates using the “spots” function. Spots can be created both manually and automatically. In our experience, automatically detecting immunopuncta of interest saves time but should be checked and corrected manually. Spots detection can be used to quantify various immunopuncta (e.g., Braude et al., 2015).

18. For automated detection of immunopuncta:
  a. Select the Spots function (yellow spheres). The spots can be renamed later.
  b. Begin automatic creation on by clicking on the blue continue arrow on bottom of the wizard menu.
  c. Select the appropriate channel.
  d. Set the “estimated x-y diameter” appropriately. (For example, 7 μm for detection of hair cell nuclei or 0.6 μm for detection of CTBP2 immunopuncta. Exact diameters for best detection will need to be adjusted for the specific points of interest.)
  e. Leave background subtraction on (checked).
  f. After continuing, a pre-selection of spots will be displayed. Various criteria (“filters”) can be used to include/exclude spots by adjusting the slider. Spots can also be included/excluded later manually as described below.
  g. Press the double green arrow to complete the spots detection.
  h. Be sure to rotate the space and zoom in and out when manually verifying all spots.

***Note:*** If the immunopuncta are not round (but, for example, oblong) the x-y diameter should be adjusted for the smaller diameter. To measure the diameter (length) of the immunopuncta of interest, go to slice mode and click on one end of the object and then the next end. The length of the line created will appear on the far-right corner of the screen.

19. For manual detection of immunopuncta:
  a. Select the Spots function (yellow spheres). The spots can be renamed later.
  b. Skip the automatic creation.
  c. Go to Pencil and select the appropriate channel.
  d. Toggle to the select mouse pointer (single arrow) by pressing Esc. Then hold the Shift Key while left clicking the immunopunctum of interest. Spots can be removed by left clicking for a second time on that spot. The size, shape, and color of the spot can be adjusted from the spots and color wheel panels.

20. Creating volumes for immunopuncta of interest with the “surfaces” function
  Surfaces of immunopuncta can be created for determination of both number and volume (e.g., Maison et al., 2013, Rohmann et al., 2015).
  a. Select the Surfaces function (blue blobs). The surface can be renamed later.
  b. Begin automatic creation by clicking on the blue continue arrow on bottom of the wizard menu.
  c. Select the appropriate channel.
  d. Select the appropriate surface level detail (generally one-tenth the approximate diameter of the immunopuncta of interest).
  e. Under threshold, use either absolute intensity or background subtraction. (When fitting surfaces, test these parameters to get the best fit.)
  f. Continue the creation wizard by clicking the blue arrow forward. Before completing the surface, it is possible to step back through the wizard and make adjustments.
  g. Adjust the threshold slider to achieve the best fit of the surface to the immunopuncta of interest. Various criteria (filters) can be used. “Quality” is generally the most helpful. Consistency between samples is critical. Surfaces that are not well fit can be excluded later. Touching objects can be split by enabling “split touching” during the creation wizard. The seed point diameter for splitting touching objects can also be adjusted.
  h. Continue the creation wizard by clicking the blue arrow forward. A pre-selection of surfaces will appear. Again, various filters can be used to include/exclude surfaces, including the number of voxels, volume, and area.
  i. Press the double green arrow to complete the surfaces detection.
  j. Surfaces can be excluded. Briefly, go to Pencil. Toggle to the select mouse pointer (single arrow) by pressing Esc. Remove a surface by left clicking on that surface and then selecting delete. The size, shape, and color of the surface can be adjusted from the surface and color wheel panels.
  k. Be sure to rotate the space, zoom in and out, and turn on and off the display channel when manually verifying the fit of all surfaces.

21. Exporting data for subsequent analysis
  a. Select the spots or surfaces of interest.
  b. Go to Statistics (graph icon)
  c. Select information to export by clicking the tools icon (left corner). Typically, the spot ID, spot x, y, z coordinates, total number of spots and/or volumes of surfaces are selected.
  d. Select the single floppy disk icon to export data to an Excel file.
  e. Be sure to verify the correctness of the information exported and then save the Excel file with the relevant identifying information that might otherwise not be exported.

### Calculation of Euclidean distances between sets of immunopuncta using custom code

**Timing: 10 min per region of interest**

This R script calculates the Euclidean distances between neighboring immunopuncta. Specifically, it calculates the distance between a given immunopunctum and its closest neighboring immunopunctum. These data (along with immunopuncta IDs) are exported as an Excel file (.xlsx format) for further analysis in R. This protocol requires Imaris (or equivalent software for calculating x,y,z coordinates for immunpuncta), Excel and RStudio. The script and complete documentation are provided in the key resources table. This script has been used previously (Sadeghi et al., 2014, Ye et al., 2017, Barone et al., 2019).

### Pillar-modiolar classification and comparison of immunolabeled inner hair cell afferent synapses using custom code

**Timing: 10 min per region of interest**

This R script pairs pre- and postsyanptic elements and classifies synapses as pillar or modiolar. Specifically, it pairs immunopuncta by calculating 3D Euclidean distances between pre- and postsynaptic volumes. It then classifies the paired volumes as pillar or modiolar by calculating a plane that defines the inner ear hair cell pillar-modiolar axis. Data are plotted in 3D figures and exported as an Excel file (.xlsx). This protocol requires Imaris (or equivalent software for calculating x,y,z coordinates and volumes for immunopuncta), Excel and RStudio. The script and complete documentation are provided in the key resources table. This script has been used previously (Barone et al., 2019, Reijntjes et al., 2020).

## Limitations

The most significant limitation results from damage to the organ of Corti, most often during isolation. Depending on the degree of damage, the organ of Corti may still be useable for quantitative image analyses. Another significant limitation is the lack of suitable antibodies or other markers for structural visualization. Tips to overcome these limitations are provided in the troubleshooting section below.

## Troubleshooting

Most problems arise from difficulty optimizing isolation of the organ of Corti and identifying reliable antibodies for immunofluorescent detection.

### Problem 1

Inner and especially outer hair cells are lost during isolation of the fixed organ of Corti from the inner ear (steps 13**–**21 in “Isolation of the organ of Corti”).

### Potential solution

Removing the lateral wall without disrupting the organ of Corti is generally more difficult basal compared to apical turns and older comapred to younger animals. Keeping the forceps between the lateral wall and the overlying bone minimizes forceful detachment of the lateral wall and prevents damage to the organ of Corti and hair cells. Additionally, the lateral wall can be cut rather than peeled away after isolation of the organ of Corti using a small dissecting knife or microscissors. In some cases, leaving small sections of the lateral wall attached may be preferable to trying to completely remove it. Spotty loss of inner and outer hair cells can occur naturally as part age-related hearing loss. Nevertheless, with practice, organs of Corti and regions of intact inner and outer hair cells can be successfully obtained from mice up to two years of age and other even older rodents (Barone et al., 2019).

### Problem 2

The organ of Corti breaks into many, smaller fragments when isolated from the inner ear, making reconstruction for tonotopic analysis difficult or impossible if segments are lost (Note in step 19 “Isolation of the organ of Corti”). If imaging precise tonotopic regions is not necessary, then this issue may not be a problem.

### Potential solution

To prevent the organ of Corti from breaking into smaller fragments, especially when removing it from the modiolus, we find that it is best to lift the organ of Corti away from the basal end of the modiolus first, then from the apical end, and then incrementally from the basal and apical ends until the middle turn is reached and detached. It is important to work slowly, especially in older animals in which the bone will be more brittle. In some cases, small fragments of the modiolus may remain attached to the isolated organ of Corti. These can be removed easily with forceps from the isolated organ of Corti. To better preserve the spiral ganglion cells, scissors can be used to first cut the centrally going projections before removing the organ of Corti from the modiolus. If, despite these tips, the organ of Corti breaks into many small fragments but the relative tonotopic locations are known, fragments can be transferred to separate wells labeled with the relative tonotopic position.

### Problem 3

During overnight incubation in the hybridization oven for RNAscope, the hybridization solution leaks across the hydrophobic barrier (Note in step 32 “Fluorescence in situ hybridization in the organ of Corti with RNAscope”).

### Potential solution

During overnight incubation, we notice that the hybridization solution can flatten and expand considerably. To avoid this problem, leave space between the solution and hydrophobic barrier and use the least amount of hybridization solution possible (≈ 50 μL; Figure 2B). To avoid cross contamination of different hybridization solutions, use one slide per hydrophobic barrier.

### Problem 4

No or non-specific immunoreactivity observed (Figure 8) (Note in step 55 “immunofluorescent staining of fixed organs of Corti”).

### Potential solution

In our experience, loss of immunoreactivity is most likely the result of decalcification and over-fixation. This protocol requires no decalcification and minimal fixation. In our experience, a minimum fixation time of 30 min in 4% PFA is sufficient. We typically fix isolated inner ears for 1 h in 4% PFA with good preservation of both tissue structure and immunoreactivity. Some antibodies prefer longer fixation times and optimal conditions should be determined experimentally. Additional steps, including antigen retrieval, have been described for improving immunoreactivity in isolated organs of Corti (e.g., McGovern et al., 2019). However, in our experience, these treatments offer only limited improvement in immunoreactivity. Testing alternative antibodies, if possible, is another potential solution. As an alternative to immunostaining, various transgenic mouse lines exist in which target proteins are genetically labeled (e.g., Figure 5).

### Problem 5

**Figure 9:**
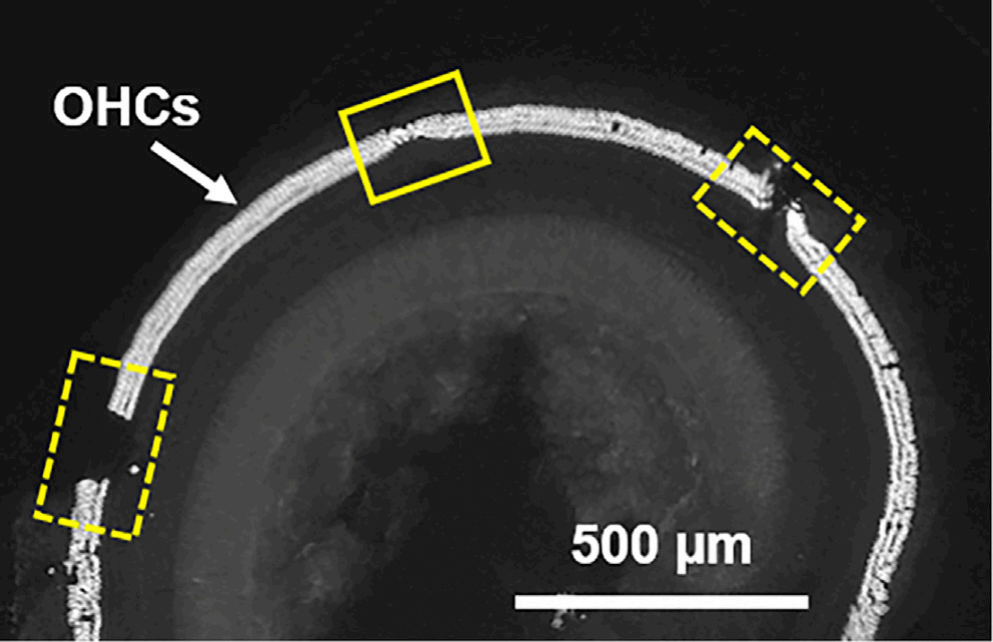
Regions of the organ of Corti are damaged or missing or not mounted flatly (Problem 5) A higher magnification epifluorescent micrograph reveals the three rows of outer hair cells (OHCs) immunolabeled with rabbit anti-prestin. The dashed boxes show regions where OHCs are missing. The solid box marks a region where OHCs are not mounted flatly.

There is difficulty with image analyses because the organ of Corti is obscured by a bubble (Figure 3) and/or not mounted flatly (Figure 9) (CRITICAL Note in step 63 “mounting of prepared organs of Corti”).

### Potential solution

For many of the image analysis protocols described, the sensorineural structures must be mounted as flatly as possible. Air bubbles while mounting are generally not a problem but are most likely to occur when small fragments of the bony modiolus remain attached to the organ of Corti. To avoid air bubbles, be sure to remove any bony debris, use sufficient mounting medium, and lower the coverslip parallel to the slide as opposed to an angled approach. Vertical placement of the slide minimizes movement of the organs of Corti during mounting.

In our experience, cold (refrigerated) organs of Corti, which are less pliable, mount more easily and more flatly (see Note in step 55). If, however, a region of the organ of Corti is not flat, imaging a neighboring region of the organ of Corti (which is mounted flatly) may be the best solution.

## Resource availability

### Lead contact

Further information and requests for resources and reagents should be directed to and will be fulfilled by the lead contact, Sonja J. Pyott (s.pyott@umcg.nl).

### Materials availability

This study did not generate new unique reagents.

### Data and code availability

Original data for figures are available from the corresponding author on request. Refer to https://github.com/thepyottlab/euclidean-distances and https://github.com/thepyottlab/pillar-modiolar for custom code for Rstudio (also provided in the key resources table above).
